# Melanoma metastatic to the hyoid bone

**DOI:** 10.1002/ccr3.3571

**Published:** 2020-11-23

**Authors:** John F. Ryan, Deborah X. Xie, Danielle F. Eytan, Edward F. McCarthy, Rajarsi Mandal, Christine G. Gourin, Evan J. Lipson, Christian F. Meyer, Peter S. Vosler

**Affiliations:** ^1^ Department of Otolaryngology–Head and Neck Surgery Johns Hopkins University School of Medicine Baltimore MD USA; ^2^ Department of Pathology Johns Hopkins University School of Medicine Baltimore MD USA; ^3^ Department of Oncology Johns Hopkins University School of Medicine Baltimore MD USA; ^4^ Johns Hopkins Bloomberg‐Kimmel Institute for Cancer Immunotherapy Kimmel Cancer Center Baltimore MD USA

**Keywords:** cutaneous malignancy, Ear, head and neck neoplasm, hyoid bone, melanoma, metastasis, nose and throat, oncology

## Abstract

Metastatic melanoma may be included in the differential diagnosis of hyoid masses in patients with a history of melanoma. Hyoid resection is well tolerated and of diagnostic and therapeutic benefit in patients with tumors metastatic to the hyoid bone.

## Graphical abstract

Metastatic melanoma may be included in the differential diagnosis of hyoid massess in patients with a history of melanoma. Hyoid resection is well tolerated and of diagnostic and therapeutic benefit in patients with tumors metastatic to the hyoid bone.

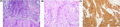

## 1 INTRODUCTION

Malignant tumors of the hyoid bone are rare and include sarcomas, plasmacytomas, and distant metastases.^1^ Only a few cases of tumors metastatic to the hyoid bone have been reported in the literature, including carcinomas originating in breast, kidney, liver, colon, and lung.^1^

Cutaneous melanoma is a malignant tumor originating from melanocytes and is the 5th most common cancer in men and 6th most common in women in the United States.^2^ Bone metastases from melanoma are relatively common, observed in 11%‐17% of melanoma patients clinically and in 23%‐49% of melanoma patients in autopsy studies. However, bony metastases usually occur in the axial skeleton, most commonly the spine.^3^ Herein, we describe the first report of melanoma metastatic to the hyoid bone.

## 2 CASE REPORT

**Figure 1:**
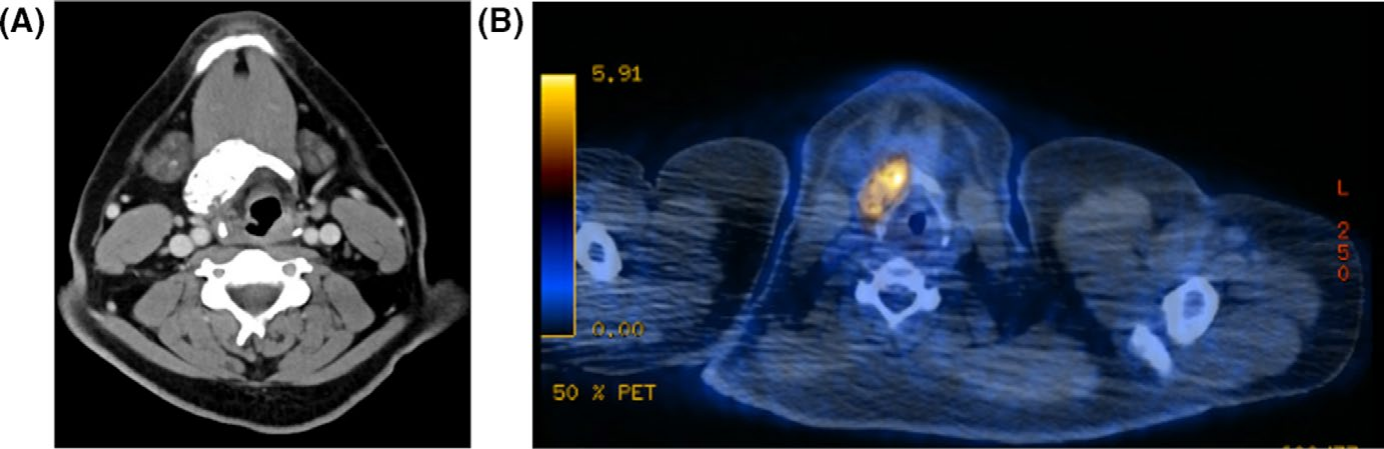
Axial imaging demonstrating a hyoid mass. A, Axial computed tomography (CT) image at the level of the hyoid bone demonstrating an expansile bony lesion within the hyoid measuring 4.1 × 2.3 × 2.8 cm. B, Positron emission tomography (PET)/CT image showing an FDG‐avid mass located within the hyoid bone measuring 4.3 × 2.0, which corresponds to the expansile mass seen on CT scan

A 27‐year‐old man presented in 2012 with a friable, pigmented lesion on his left posterior neck that bled with palpation. A biopsy demonstrated melanoma with Breslow thickness of 5 mm, and treatment included a wide local excision with 2‐cm margins with no sentinel lymph node biopsy or adjuvant therapy. Six years later, the patient developed hemoptysis and was found on computed tomography (CT) scan of the chest to have a 3.1 × 2.7 × 3.6 cm mass in the lower lobe of the right lung. Fine‐needle aspiration of the mass performed under endobronchial ultrasound guidance demonstrated a malignant small round blue cell tumor. Molecular and genetic studies were consistent with an EWSR1 translocation negative Ewing sarcoma/primitive neuroectodermal tumor (PNET). A positron emission tomography (PET)/CT scan revealed an FDG‐avid right lower lobe mass and an intensely FDG‐avid and partially calcified mass on the right hyoid bone (Figure 1). The patient was referred to the otolaryngology service for evaluation of the hyoid lesion.

**Figure 2:**
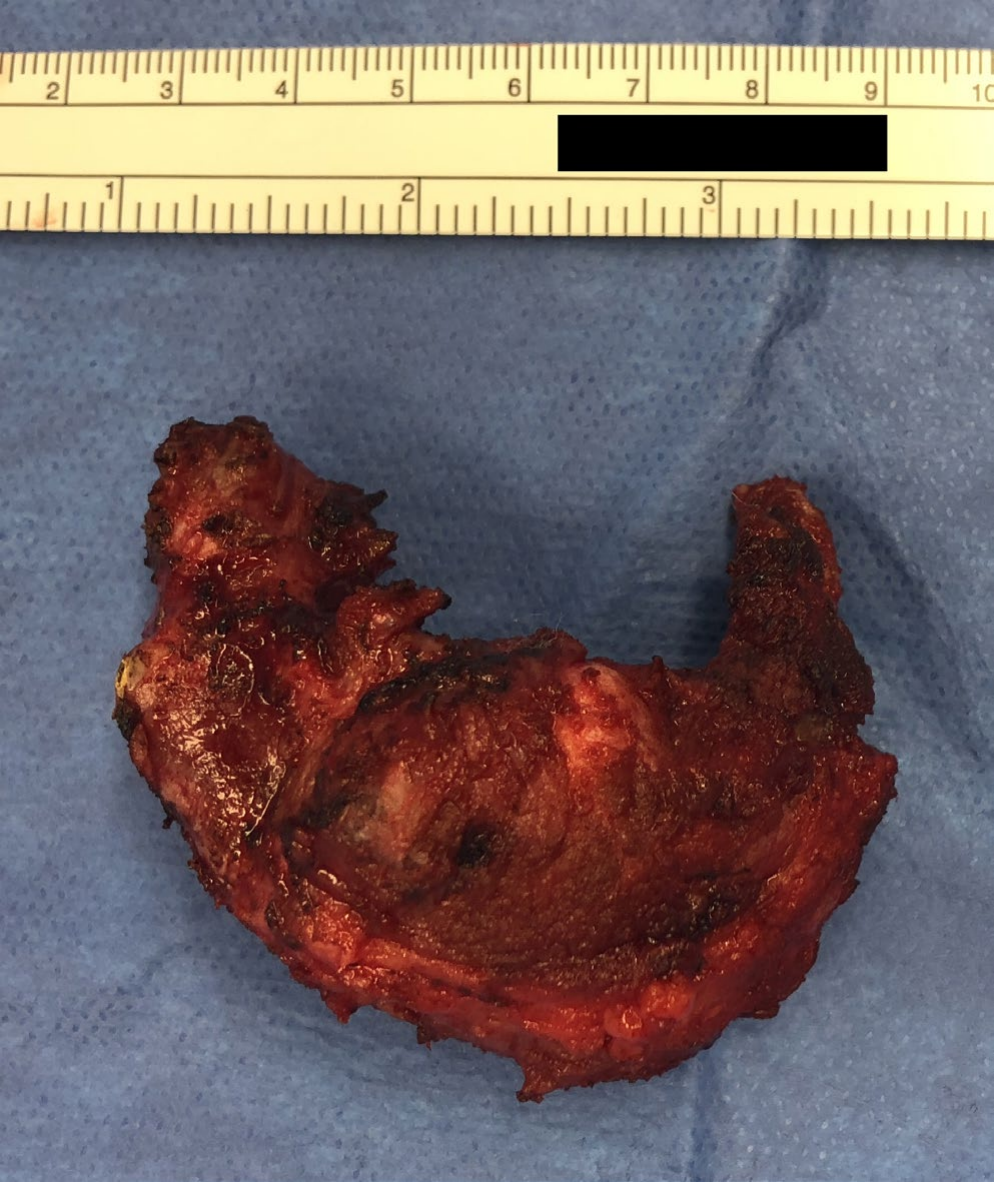
Partial hyoid bone resection gross specimen. The hyoid bone measured 6.5 × 5.5 cm. The bone was resected by detaching the supra‐ and infra‐hyoid musculature circumferentially around the bone to preserve the muscles and protect adjacent neurovascular structures including the hypoglossal nerve

**Figure 3:**
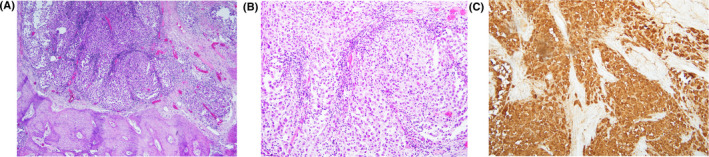
Histopathologic analysis of the tumor specimen demonstrated (A) melanoma cells within the hyoid bone with associated dense reactive bone formation on low power microscopy, (B) sheets of melanoma cells on high power microscopy, and (C) S100 positivity of the melanoma cells on immunohistochemical staining

Visualization of the lesion by ultrasound was technically challenging secondary to its location within bone. A core needle biopsy of the mass was nondiagnostic, showing only rare atypical epithelial cells in a background of lymphocytes. Consensus from a multidisciplinary sarcoma tumor board review was that the hyoid lesion likely represented a synchronous primary tumor (eg, plasmacytoma, chondrosarcoma, osteosarcoma). Given the severity of the patient's pulmonary symptoms including hemoptysis and dyspnea, standard‐of‐care chemotherapy (14 cycles of vincristine, doxorubicin, cyclophosphamide, ifosfamide, and etoposide (VCD/IE)) was initiated. It was thought that both the lung mass and hyoid tumor might respond to chemotherapy. Mid‐treatment PET/CT imaging demonstrated substantially decreased size and complete metabolic response of the lung mass but development of right neck adenopathy and growth of the hyoid bone mass, concerning for a separate malignancy. Four months after initiating chemotherapy, he underwent surgical resection of the hyoid mass and a selective neck dissection. Approximately 75% of the hyoid bone was resected including a 1‐cm margin of normal appearing bone away from the mass (Figure 2). Pathology of the hyoid mass demonstrated metastatic melanoma with tumor identified less than 0.1 cm from the resection margin (Figure 3). The neck dissection specimen included 21 lymph nodes, all negative for malignancy.

He completed his VCD/IE chemotherapy with resolution of the pulmonary tumor. Completion hyoid bone resection was performed to obtain clear margins; pathology evaluation revealed no residual malignancy. He recovered well functionally after his surgeries tolerating a regular diet without evidence of dysphagia. Postoperatively, he began adjuvant immunotherapy with nivolumab for metastatic melanoma treatment. Imaging performed 15 months after initial hyoid mass resection demonstrated no evidence of neoplasm.

## 3 DISCUSSION

The most common sites of distant melanoma metastases are skin, lung, brain, liver, bone, and intestine.^3^ In the head and neck, melanoma frequently metastasizes to cervical lymph nodes and may present as a neck mass.^3^ However, melanoma metastatic to the hyoid bone has not been reported. In this case, given the patient's diagnosis of Ewing sarcoma/PNET and remote history of melanoma, the primary diagnoses considered were metastatic Ewing sarcoma/PNET and a tumor primary to the hyoid bone, including plasmacytoma, chondrosarcoma, or osteosarcoma. Surgical resection was performed in this case for both diagnostic and therapeutic purposes once a mixed response to VCD/IE therapy was observed between the lung and hyoid lesions.

Melanomas may mimic the histologic features of a wide variety of tumors including lymphomas, poorly differentiated carcinomas, neuroendocrine tumors, sarcomas, and germ cell tumors and can have such diverse cytoplasmic morphologies as clear cell, signet ring shape, rhabdoid, ballooning, and plasmacytoid appearance.^4^ Immunohistochemical stains are therefore used to differentiate melanoma from other tumors. Melanoma may stain positive for S‐100, HMB‐45, MART‐1/Melan‐A, tyrosinase, Sox10, and MITF.^4^ In this case, the neoplasm identified in the hyoid bone was positive for S‐100, Melan‐A, HMB‐45, and Sox10 and negative for AE1/3, CD68, CD1a, and Langerin, consistent with melanoma.

The hyoid bone serves as a site of attachment for tongue, pharyngeal, and neck muscles and functions in coordinating swallowing and speech. Hyoid resection may result in dysphagia given destabilization of these muscular attachments. Concern for debilitating dysphagia was the impetus for partial hyoid resection initially. Fortunately, no complications from hyoid excision were observed following either procedure. The favorable functional outcome in this case may be credited to young age and otherwise relatively good health, serving as an illustration that hyoid resection can be well tolerated in select patients.

Immunotherapy has become a standard of care in the treatment of advanced melanoma, and nivolumab, a fully human monoclonal antibody against PD‐1, has demonstrated excellent antitumor activity and favorable safety profile with durable responses in metastatic melanoma.^5^ Due to excellent antitumor effect and limited toxicity profile compared to traditional radiation and chemotherapy, nivolumab alone was chosen for adjuvant therapy with no evidence of melanoma relapse.

## 4 CONCLUSION

In summary, our report suggests that metastatic melanoma should be included in the differential diagnosis for any patient who presents with a hyoid tumor and has a prior history of melanoma. Hyoid bone resection may be considered in patients with hyoid tumors given ease of exposure, diagnostic, and therapeutic benefit, and the potential for an excellent functional outcome postoperatively.

## CONFLICT OF INTEREST

None declared.

## AUTHOR CONTRIBUTIONS

JR performed chart review of patient case and wrote the manuscript. DX, DE, EM, RM, CG, EL, CM, and PV provided critical review and contributed to the writing of the manuscript. EM provided figures for the manuscript. JR and PV conceived the idea of the manuscript. All authors discussed the conclusions and contributed to the final manuscript.

## ETHICAL APPROVAL

The patient provided consent for the publication of this report. IRB Approval: This study was considered exempt from IRB review as a case report.

## Data Availability

All data pertinent to this study beyond that available in the published article are available from the corresponding author on reasonable request.
